# Demographic and Attitudinal Factors of Adherence to Quarantine Guidelines During COVID-19: The Italian Model

**DOI:** 10.3389/fpsyg.2020.559288

**Published:** 2020-10-21

**Authors:** Leonardo Carlucci, Ines D’Ambrosio, Michela Balsamo

**Affiliations:** School of Medicine and Health Sciences, University of Studies “G. d’Annunzio” Chieti–Pescara, Chieti, Italy

**Keywords:** adherence, risk perception, quarantine, confinement, coronavirus disease 2019

## Abstract

In Italy, a large outbreak of coronavirus disease 2019 (COVID-19) occurred from 2020 January 30, before the World Health Organization has stated that it is a pandemic. The nationwide quarantine had the desired impact of controlling the epidemic, although had presented many challenges, given its large economic and social costs. Complete adherence to recommendations can potentially decelerate and reduce infectious disease outbreaks. To date, it is not clear how compliant the Italian public has been with voluntary home quarantine, neither which factors have influenced an individual’s decision to comply with a quarantine order. The purposes of this study were to investigate the degree of the adherence to quarantine restrictions and the factors associated with the self-reported adherence. During the third week of the national lockdown, 3,672 Italian quarantined adult residents (65% females; range, 18–85 years) participated in an online cross-sectional survey focused on the risk perception of contracting COVID-19 and their reported adherence to quarantine protocols. Analysis of variance showed significant differences among demographic groups in tendency to comply with quarantine orders, with women, most educated people, residents of Southern Italy, middle-aged individuals, and health workers more likely to adhere to quarantine guidelines. As well, participants exhibiting the perception, anxiety, and susceptibility of risk of contracting COVID-19 disease were found significantly more likely to adhere to quarantine guidelines. The results of this study can help public health policy makers to recognize target populations for COVID-19 prevention and health education and to understand how inform communication strategies aimed at minimizing the impact and spread of the disease.

## Introduction

The novel coronavirus disease 2019 (COVID-19) had gained intense attention globally and continues to spread, posing a serious human pandemic threat (World Health Organization, 2020b). Given the lack of the proven vaccine, or efficacious treatments for infected people, the “killer” virus is arousing the sense of danger and uncertainty of its future course among health workers and the public.

After China, Italy has the second largest number of confirmed cases and was the first Western Republic affected by the COVID-19 spread (Saglietto et al., 2020). The Italian National Institute of Health reported that between December 2019 and April 16, 2020, approximately 22,170 deaths occurred, 40,164 people were discharged or healed, and 168,941 million people were infected with COVID-19 on 1,046,910 tampons performed. In response to the rising numbers of suspected and diagnosed cases and deaths and to maintain the capacity of health systems to treat as many severe cases as possible, in Italy and in the world, a range of control measures had urgently adopted or are in the process of implementing, such as “isolation” and “quarantine,” as non-pharmaceutical interventions tools to slow or prevent spread (Schabas, 2004; Bensimon and Upshur, 2007). The Italian government declared the national lockdown status, by March 11 to May 3: all public places were closed, and people have to stay at home apart from exercise, serious health issues, and other essential tasks (Government of Italy, 2020b). All the Italian people were in quarantine (#iorestoacasa).

Punitive legislation for travelers who make false health declarations and/or ignore these recommendations was established. Similar to pharmaceutical interventions, the effectiveness of quarantine interventions should be evaluated and monitored over time.

According to the Imperial College COVID-19 Response Team (Ferguson et al., 2020), the slowing growth in daily reported deaths was consistent with a significant impact of the containment and quarantine measure implemented several weeks earlier. The effective reproduction number, Rt, was dropped to close to 1 around the time of lockdown (March 11). This meant that 38,000 (13,000–84,000) deaths were averted.

At the time of writing, these measures had been extensively followed in public health of most nations, with a substantial impact in reducing transmission in countries with more advanced epidemics (Ferguson et al., 2020).

Successful use of these major non-pharmaceutical interventions requires a good organization of health services and mostly a good adherence to protocols by citizens. As Webster et al. (2020) stated, “Quarantine does not work if people do not adhere to it” (p. 3). However, little is known about which factors can increase the likelihood of general population adhering to quarantine orders in a Western republic, like the Italian one (Gernhart, 1999).

During major epidemics, social variables (gender, age, ethnicity, education level, marital status, working status) were found associated to self-quarantine guidelines (Bish and Michie, 2010; Webster et al., 2020). In addition, a psychological factor affecting the adherence to quarantine was identified in beliefs about potential health-specific harm, such as “risk perception.” Risk perceptions are placed as core concepts in most theories of health behavior, including the Protection Motivation Theory (Rogers, 1975), the Health Belief model (Rosenstock, 1974), Theory of Planned Behavior (Ajzen, 1991), and the Extended Parallel Process model (Witte, 1992; for reviews, see Sutton, 1987; Weinstein, 1993, 2000).

Indeed, perceived likelihood, susceptibility, or severity has been found to shape or predict many health behaviors. In many studies, these dimensions of risk perception were associated with the compliance with physician-prescribed medical regimens, such as the vaccination behavior [for a meta-analysis, see (Brewer et al., 2007)], adherence to measures for preventing transmission of microorganisms in primary healthcare (Maroldi et al., 2017), adherence to effective measures in preventing HIV infection (Storholm et al., 2017), and adherence to mammography guidelines as screening for women at risk of breast cancer (Graves et al., 2008). Also, during past epidemics, such as Ebola, perceived risk has been found to both positively and negatively influence health behaviors (Bish and Michie, 2010; Ajilore et al., 2017).

In addition, according to the literature on risk perception (Adams, 1995), public concerns about risk are higher with novel threats and, when individuals do not feel in control of the risk, both factors relevant to an influenza pandemic. There is also evidence from this review that perceiving the disease to be more severe is associated with taking preventive and avoidant behaviors. This is the case of the COVID-19 emergency.

All the aforementioned theories highlighted the importance of perceptions of threat in determining a behavioral response and provide a framework to understand the findings of these studies.

In light of this literature and the advent of this current influenza pandemic, the purpose of this study was to investigate (1) the degree of the adherence to quarantine restrictions and recommendations in a large sample of quarantined Italian participants during the COVID-19 pandemic and (2) the factors associated with the self-reported adherence to these measures (or protective behaviors).

Based on the previous literature (Bish and Michie, 2010; Webster et al., 2020), sociodemographic variables, such as gender, age, education, health, and marital status, employment status and characteristics, and the risk perception (perceived risk of contracting COVID-19) were selected as factors that could influence the self-reported compliance with the quarantine guidelines.

In addition to sociodemographic variables, geographical region of residence was examined (North-east, North-west, Center, South, Islands). The regional structure of the Italian National Health Service translated into very diverse responses to the emergency from the regions, which have differentiated, by spread of the epidemic, the number of diagnosed cases and deaths (Remuzzi and Remuzzi, 2020; Spina et al., 2020). Participants from different countries are hypothesized to show different values in perceived risk of contracting COVID-19 and adherence to quarantine guidelines.

## Materials and Methods

### Sample and Procedure

Respondents selected for this study were quarantined Italian adults 18 years or older with access to a networked computer. An online cross-sectional study was conducted using a virtual snowball sample through social media. The study has been recorded to the Ministry of Education, University and Research and approved by the Department of Psychological Sciences, Health and Territory, University of Studies “G. d’Annunzio” Chieti–Pescara, Italy, review board. Written informed consent was obtained from all individual participants included in the study. This cross-sectional survey was conducted between March 21 and 26, 2020, the 2 weeks immediately after the lockdown was decreed by the Italian Government on March 9 (#iorestoacasa) (Government of Italy, 2020a). We received responses from 3,964 respondents. Of these, 292 respondents did not complete the questionnaires (>50% of the missing values).

### Measures

#### Sociodemographic Variables

General information, sociodemographic variables (such as age, education, marital status, geographic area and region, employment status, and year income) including history of psychiatric illnesses and medical problems (e.g., physical/mental pathologies; hospitalizations), and diagnosis or suspect of COVID-19, as well as living environment during quarantine, were asked (e.g., household type and size). In addition, participants were asked to rate their physical symptoms during quarantine (e.g., fever, cough, difficulty in breathing). Participants’ physical health status index was derived from history of chronic medical illness and number of pathologies (none = excellent, 1 pathology = good, 2 pathologies = poor, ≥3 pathologies = fragile). Questions about religious practices and religiousness were also included in the survey.

#### Primary Outcome

Adherence to quarantine guidelines carried out in response to COVID-19 infection was measured by 12 items classified into three categories: preventive, avoidant, and management of disease behaviors (Bish and Michie, 2010). The preventive behavior category was composed of six items, which include hygiene behaviors such as handwashing with soapy water or an alcohol-based solution, coughing or sneezing into a handkerchief (preventing the hands from coming into contact with respiratory secretion), cleaning surfaces with chlorine or alcohol disinfectant, wearing protective mask, keeping at least 1 m (or 3 feet) of distance. The avoidant behaviors category included five questions about the avoidance of gatherings in public or open-to-public places, handshaking and hugging, touching eyes/nose/mouth with hands, sharing of promiscuous use of glasses and bottles, doing and outdoor sport, and/or physical activities alone in public areas (Appendix A). Respondents were asked about the frequency whether they had carried out quarantine guidelines on a 5-point Likert scale (from 0 “never” to 4 “always”), respectively, for the preventive and avoidant categories. A global index of adherence to quarantine guidelines has been developed, by summing the answers to the items below. These behaviors are all decreed by law (Government of Italy, 2020a). In the present sample, Cronbach α was 0.696 [95% confidence interval (CI), 0.681–0.710].

Finally, a single item about the “taking antiviral drugs” has been included in the questionnaire as a management of disease behaviors category.

#### Risk Perception

A multidimensional questionnaire on risk perception of COVID-19 infectious disease outbreak has been implemented following the “Effective Communication in Outbreak Management” guidelines and using a standardized and revised version of the Ebola risk perception surveys (Richardus et al., 2015; see Appendix A). The risk perception questionnaire contains the following dimensions: (A) perception (eight items); (B) anxiety and susceptibility to the COVID-19 (three items); (C) intention to carry out the preventive measures (one item); (D) perception of seriousness (one item); and (H) motivating/hindering factors that determine the willingness to carry out preventive measures (one item). Risk questions were responded to on a 5-point Likert scale for the dimensions A to D, whereas for H, response options were in a multiple-choice format. In addition, perception (A) risk dimension was classified into four groups according to quantiles (very low, low, high, and very high). In the present sample, Cronbach α for the risk perception dimension was 0.808 (95% CI, 0.798–0.817).

### Data Analysis

Descriptive statistics were calculated for sociodemographic characteristics, physical symptoms and health service utilization variables, contact history variables, knowledge and concern-related variables, precautionary measure variables, and additional health information variables. A series of independent-samples *t* test and analyses of variance (ANOVAs) were carried out to determine whether there is a statistically significant difference (*p* < 0.05) in the adherence to quarantine guidelines scores between levels of risk perception and demographic factors. Statistical analysis was performed using SPSS Statistic 21.0 (IBM SPSS Statistics). Effect sizes (ESs) for independent *t* test was calculated using the Hedges *g*, in order to provide a measure of ES weighted according to the relative size of each sample. The Hedges *g* ES was interpreted using Cohen (1988) convention as small (0.2), medium (0.5), and large (0.8). ESs for the ANOVAs were computed using the partial ω^2^ (ωp^2^). Partial ω^2^ represented an unbiased alternative to partial η^2^ (Olejnik and Algina, 2003). ωp^2^ was interpreted according to Murphy et al. (2014) convention as small (0.01), medium (0.06), and large (>0.15).

## Results

### Sociodemographic Data

Sociodemographic variables and levels of risk perception are shown in Table 1. More than half of the participants were females (65.1%), with an average age of 33.27 (SD = 14.29) years and with a high level of education (49.5% upper secondary school, 41.8% bachelor’s degree), single (61.2%), Roman Catholic (73.2%), and located in the South of Italy (45.2%). The 31.20% were students, and the 47.4% of the participants were employed, with a yearly income of 10,000–30,000 €. Among these, 5.2% declared they had moved from one to another city in the previous weeks, due to pandemic. The 6.3% of our sample declared to be employed as healthcare professionals: 20.3% were physicians, 14.7% were nurses, and 4.7% were pharmacists.

**TABLE 1:** Descriptive and differences among sociodemographic variables and risk in adherence to quarantine Guidelines (*n* = 3,672).

Group		Descriptive		Adherence to quarantine guidelines	
Sex Variables	Frequency	%	Mean	SD	
	Man	1,282	34.9	31.24	5.61	
	Women	2,390	65.1	33.32	4.91	
*t*(3670) = −11.145, *p* < 0.001, Hedges *g* = 0.401					

**Age (years)**	**Frequency**	**%**	**Mean**	**SD**	***Post hoc***

(1) 18–29	1,995	54.3	31.50	5.41	1 vs. all 2 vs. 4
(2) 30–39	723	19.7	33.38	4.47	
(3) 40–49	404	11	34.17	4.89	
(4) 50–59	261	7.1	34.92	3.98	
(5) >60	289	7.9	33.84	5.64	
*F*(4;3667) = 54.334, *p* < 0.001, ω*p*^2^ = 0.054					

**Education**		**Frequency**	**%**	**Mean**	**SD**	

(1) Primary school	56	1.5	31.18	8.23	4 vs. all
(2) Lower secondary school	264	7.2	31.83	5.76	
(3) Upper secondary school	1,817	49.5	32.30	5.35	
(4) Bachelor/master/doctorate	1,535	41.8	33.12	4.86	
*F*(3;3668) = 10.228, *p* < 0.001, ω*p*^2^ = 0.007					

**Marital status**		**Frequency**	**%**	**Mean**	**SD**	

(1) Single	2,249	61.2	31.83	5.33	1 vs. 2, 3, 4 4 vs. 2, 3
(2) Married	901	24.5	34.07	4.84	
(3) Divorced/separated	103	2.8	34.81	4.38	
(4) Cohabiting	366	10	33.00	4.85	
(5) Widowed	53	1.4	32.83	6.42	
*F*(4;3667) = 36.097, *p* < 0.001, ω*p*^2^ = 0.036					

**Geographic area**		**Frequency**	**%**	**Mean**	**SD**	

(1) North-West	897	24.4	32.02	5.20	2 vs. 3 4 vs. 1, 2, 3
(2) North-East	338	9.2	31.33	5.20	
(3) Central	522	14.2	32.39	5.56	
(4) South	1,660	45.2	33.23	5.17	
(5) Islands	165	4.5	32.60	4.95	
	*Missing*	90	2.5			
*F*(4;3577) = 13.96, *p* < 0.001, ω*p*^2^ = 0.014					

**Health Status**		**Frequency**	**%**	**Mean**	**SD**	

(1) Excellent	2,616	71.2	32.47	5.26	4 vs. 3
(2) Good	622	16.9	33.07	4.88	
(3) Poor	125	3.4	33.66	5.83	
(4) Fragile	24	0.7	30.63	8.83	
*Missing*	285	7.8				
*F*(3;3383) = 5.023, *p* = 0.002, ω*p*^2^ = 0.003					

**Employment status**		**Frequency**	**%**	**Mean**	**SD**	

(1) Unemployed	416	11.3	33.20	5.23	1 vs. 4 3 vs. all 5 vs. 4
(2) Retired	144	3.9	33.56	6.13	
(3) Student	1,138	31	31.20	5.48	
(4) Healthcare professional	232	6.3	34.97	4.09	
(5) Employed	1,742	47.4	32.96	4.95	
*F*(4;3667) = 38.073, *p <* 0.001, ω*p*^2^ = 0.038					
(1) Doctor	47	20.3	36.06	3.19	4 vs. 1, 2
(2) Nurse		34	14.7	36.35	3.70
(3) Pharmacist		11	4.7	36.36	2.76
(4) Rehabilitation Therapists		59	25.4	33.55	4.83
(5) Psychologist		69	29.7	34.28	3.98
*Missing*		12	5.2		
*F*(4;215) = 4.551, *p <* 0.001, ω*p*^2^ = 0.060					

**Income per year**		**Frequency**	**%**	**Mean**	**SD**	

0–10,000 €		502	13.7	33.06	5.09
10,000–30,000 €		1,193	32.5	33.10	4.86
30,000–50,000 €		214	5.8	33.69	4.76
>50,000 €		65	1.8	34.23	4.13
Missing		1,698	46.2		
*F*(3;1970) = 1.970, *p* = 0.116					

*North-West (Piemonte, Lombardia, Liguria, Valle d’Aosta). North-East (Veneto, Friuli Venezia Giulia, Emilia Romagna, Trentino). Center (Toscana, Umbria, Marche, Lazio). South (Abruzzo, Molise, Campania, Puglia, Basilicata, Calabria). Islands (Sicilia, Sardegna). *Post hoc* test = Tukey HSD. Not significant differences have been found between No Antiviral Drugs and Sociodemographic Variables, except for health status (Kruskal–Wallis = 13.347(3); *p* = 0.004).*

Most participants were in quarantine with family (84.2%), 6.5% alone, and the remaining with colleagues/roommate/other familiars. Participants lived in apartments with balcony (74.2%), approximately within 80–150 mq (52.5%).

Subjects were mostly healthy: 71.2% reported no physical pathologies, whereas 7% were detected as fragile and with a long history of chronic medical illness and several number of pathologies (e.g., diabetes or cancer, etc.). During this period of quarantine, 64.95% of the participants had reported no symptoms likely related to the COVID-19 infection, while the remaining has reported the following symptoms: cold (9.59%), feeling of weakness (8.22%), cough (7.5%), difficulty in breathing (1.44%), fever higher than 37.5°C (0.68%), and changes in taste/smell (0.14%).

### Risk Perception and Adherence to Quarantine Guidelines

Participants exhibited an average perception of risk (Risk A), with a mean of 26.05 (SD = 5.89; range of scores, 8–40). In details, the perception of risk of contracting COVID-19 disease (Risk A) was homogeneously distributed across the four risk levels, from 30.8% of the participants perceiving very low risk to 20.6% of those perceiving very high risk (Table 2). As regards *anxiety* and *susceptibility* risk dimension (Risk B), 92.4% of the participants was found worried about getting COVID-19, from slightly to really worried; 95.6% perceived the *chances of getting COVID-19* from average to very high, *if* they did not *follow preventive measures adopted*; 91.5% of the respondents reported a medium to very high probability of getting COVID-19 within the next year, if they did not get vaccinated.

**TABLE 2:** Descriptive of risk perception dimensions (*n* = 3,672).

Group	Risk dimensions	Descriptive		Adherence to quarantine guidelines		
		Frequency	%	Mean	SD	*Post hoc*
**Risk A) Perception**						
(1) Very low		1,132	30.8	32.70	5.37	4 vs. 2, 3
(2) Low		888	24.2	32.08	5.17	
(3) High		897	24.4	32.37	5.36	
(4) Very high		755	20.6	33.31	4.98	
*F*(3;3668) = 8.337, *p* < 0.001, ω*p*^2^ = 0.006						
**Risk B) Anxiety and Susceptibility**						
*H ow worried are you about getting COVID-19?* (Risk B1)						
(1) Not worried at all		44	1.2	28.41	8.38	5 vs. all
(2) Not worried		234	6.4	30.53	6.04	
(3) Slightly worried		1,068	29.1	31.35	5.19	
(4) Worried		1,540	41.9	33.03	4.90	
(5) Really worried		786	21.4	34.27	4.81	
*F*(4;3667) = 56.888, *p* < 0.001, ω*p*^2^ = 0.057						
*What are your chances of getting COVID-19 if you do not follow preventive measures adopted?* (Risk B2)						
(1) Very low		45	1.2	32.64	5.39	5 vs. all
(2) Low		117	3.2	31.59	6.14	
(3) Average		451	12.3	31.03	5.99	
(4) High		1,399	38.1	32.13	5.03	
(5) Very high		1,660	45.2	33.48	5.00	
*F*(4;3667) = 26.358, *p* < 0.001, ω*p*^2^ = 0.026						
*If you don’t get vaccinated. what are your chances of getting COVID-19 within this year?*(Risk B3)						
(1) Very low		87	2.4	32.94	5.66	5 vs. all
(2) Low		226	6.2	32.21	5.60	
(3) Average		1,488	40.5	32.30	5.29	
(4) High		1,355	36.9	32.66	5.11	
(5) Very high		516	14.1	33.37	5.23	
*F*(4;3667) = 4.511, *p* < 0.001, ω*p*^2^ = 0.003						
**Risk C) Intention**						
*Do you intend to follow the preventive measures?*						
	Absolutely not	5	0.1	27.20	11.30	
	Probably not	4	0.1	23.00	9.27	
	Probably yes	170	4.6	29.75	6.01	
	Absolutely yes	3,493	95.1	32.75	5.15	
**Risk D) Perception of seriousness**						
*How severe do you think the coronavirus emergency on global health is?*						
	Not serious at all	1	0.1	31.00	—	
	Not very serious	53	1.4	28.28	8.08	
	Quite serious	1,087	29.6	31.25	5.57	
	Very serious	2,531	68.9	33.26	4.88	
**Risk H) Motivating/hindering factors**						
Why are you taking protective behaviors?*						
	I want to prevent the spread of COVID-19	2,930	79.8			
	I don’t want to transmit COVID-19 to people close to me	2,779	75.7			
	COVID-19 can be dangerous	2,160	58.8			
	I feel responsible for my health	1,647	44.9			
	I trust the preventive measures are helpful	1,122	30.6			
	Authorities recommend them	370	10.1			
	I might regret it later if I don’t take them.	357	9.7			
	I think I’m at risk COVID-19	161	4.4			
	Other people at home or at work are following them already	85	2.3			
	I’m often sick	75	2			
	I don’t want problems with the law	6	0.2			
	Desire to return to normal	2	0.1			
	Civil responsibility	2	0.1			

*More than one options is possible. *Post hoc* test = Tukey HSD. No significant differences have been found between No Antiviral Drugs and Risk dimensions. Risk c-d-h dimensions contains zero/few cases in one or more category response and then discharged from mean/frequency analysis.

As regards the intention, 95.1% of the subjects expressed the willingness to carry out to follow the preventive measures without any doubt (Risk C), mostly driven from the following motivation factors (Risk H), from preventing the spread of COVID-19 (79.8%), avoiding to transmit COVID-19 to people close to me (75.7%), to considering the government actions helpful (30.6%) (Table 2). As for the perception of seriousness, 98.5% of the respondents perceived “The coronavirus emergency was quite and very serious threat on global health” (Risk D).

Information about COVID-19 emergency were sought by our respondents from the following media: 78.8% by radio or television newscasts, 69% from official channels (press releases, bulletins), 55.7% from social networks, 36.8% from newspapers (including its digital editions), and 12.8% from relatives and friends.

### Adherence to Quarantine Guidelines

Respondents exhibited medium to high scores of adherence to quarantine guidelines as measured by a single interval index, with a mean of 32.59 (SD = 5.22; range, 0–44), with an average of quarantine duration of 15 days (SD = 6.64). A weak but significant positive association (*r* = 0.035, *p* = 0.034) was found between the adherence and the length of quarantine.

Within the preventive behaviors’ category, covering the mouth or nose (when sneezing and/or coughing) and keeping at least 1 m of distance (also named social distancing measure) were the behaviors most always adopted by participants (78.5% and 70.6%, respectively), and then the use of handkerchief (54.5%), handwashing using soapy water or alcohol-based solution (40.8%), and wearing face mask (35.7%). Instead, the cleaning of surfaces represented the less frequent protective behavior (26.2% of “always” responses) of our respondents.

As avoidant behaviors, avoidance of gatherings (92.8%), of handshake/hug (76.8%), to do outdoor sports alone in public area (77.3%), and sharing of glasses and bottles (61.3%) were the most protective behaviors always adopted by our participants. Less importantly, avoidance to touch eyes/mouth with hands was adopted by 18% of the participants.

Finally, within the management of disease behaviors, only 0.9% of the participants take antiviral drugs even if not medically prescribed, and 5.4% with a medical prescription.

Reasons for going out declared by our respondents were as follows: 8.5% for working demands, 23.9% for receiving medical treatments or for going to the pharmacy, 9.7% for essential necessities (to procure groceries), 1% for assisting families, and 5% for physiological needs of own domestic animal.

### Differences in Adherence to Quarantine Guidelines

Differences among sociodemographic variables, as well as in risk perception dimensions, were found in adherence to protective measures scores. Means and standard deviations, group *F*/*t* tests, *p* values, and Tukey *post hoc* analysis were provided for each independent variable in Tables 1, 2. Pairwise deletion techniques were applied to handling missing data.

As regards sex, women exhibited significantly higher levels of adherence to quarantine guidelines [*t*(3670) = −11.145, *p* < 0.001, Hedges *g* = 0.401] compared to the men.

A statistically significant difference was found between age groups as determined by one-way ANOVA [*F*(4;3667) = 54.334, *p* < 0.001, ω*p*^2^ = 0.054]. A *post hoc* test revealed that the 18- to 29-year age group scored statistically significantly lower (*p <* 0.001) compared to other age groups. Equally, the 50- to 59-year age group was statistically significantly higher (*p <* 0.001) in adherence scores compared to the 30- to 39-year group.

Participants with a high education showed the highest scores of adherences among the other levels of education [*F*(3;3668) = 10.228, *p* < 0.001, ω*p*^2^ = 0.007].

Significant differences in adherence within the marital status [*F*(4;3667) = 36.097, *p* < 0.001, ω*p*^2^ = 0.036] revealed that singles’ (*p <* 0.001) adherence mean was statistically significantly lower than married, divorced/separated, and cohabiting status means. In addition, divorced/separated and married groups equally exhibited significant higher adherence (*p <* 0.01) compared to cohabiting people.

Differences of geographic area in adherence to quarantine guidelines [*F*(4;3577) = 13.96, *p* < 0.001, ω*p*^2^ = 0.014] revealed that participants from the Southern Italy showed statistically significantly higher level of adherence (*p* < 0.01) compared to the Central and North West/East areas, except for the Islands. Residents from Central Italy (*p <* 0.05) were statistically significantly higher in adherence levels compared to the North-East.

A slight but significant difference among adherence levels was found across health status variables (*p* < 0.01), where the fragile group (*p <* 0.05) showed the lowest mean, which was significantly lower compared to poor group.

Next, a statistically significant difference was found among the employment status [*F*(4;3667) = 38.073, *p <* 0.001, ω*p*^2^ = 0.038]; in details, students’ mean (*p <* 0.001) was statistically lower compared to the other status. As expected, healthcare professional adherence mean (*p <* 0.001) was significantly higher compared the unemployed and employed. Because the healthcare professions category was quite broad, and not all categories are employed on the frontlines, a statistically significant difference among healthcare professionals was found [*F*(4;215) = 4.551, *p <* 0.001, ω*p*^2^ = 0.060]. A *post hoc* test revealed that rehabilitation therapists’ adherence mean (*p <* 0.01) was statistically significantly lower than that of doctors and nurses. No statistically significant difference was found in adherence to quarantine guidelines for income for year groups.

A statistically significant difference between levels of risk perception A [*F*(3;3668) = 8.337, *p <* 0.001, ω*p*^2^ = 0.006] was found. A *post hoc* test revealed that the very high level of risk perceived (*p <* 0.001) was statistically significant compared to the high and low levels of risk perceived. The same trends in differences were found among risk anxiety [*F*(4;3667) = 56.888, *p <* 0.001, ω*p*^2^ = 0.057] and susceptibility levels [Risk B2, *F*(4;3667) = 26.358, *p <* 0.001, ω*p*^2^ = 0.026; and Risk B3, *F*(4;3667) = 4.511, *p* < 0.001, ω*p*^2^ = 0.003]. A *post hoc* test revealed that the last category was statistically higher compared to the previous (*p <* 0.01).

No statistical differences were found between the sociodemographic variables and antiviral drugs’ protective behavior [except for health status, Kruskal–Wallis = 13.347(3); *p* < 0.01].

## Discussion

Italy was the first Western Republic affected by the COVID-19 spread (Saglietto et al., 2020). Despite the criticism about the lack of its scientific basis (Schabas, 2004; Bensimon and Upshur, 2007; Greenberger, 2018), the slowing growth in daily reported deaths in Italy was consistent with a significant impact of quarantine implemented several weeks earlier. Successful use of quarantine as a public health measure in a democratic society requires increasing the likelihood of people adhering to protocols.

A large sample of Italian quarantined adults showed very high rates of adherence to quarantine restrictions and recommendations, after 15 days of the national lockdown, due to COVID-19 pandemic.

Differences among sociodemographic variables indicated that women were more likely to carry out protective behaviors compared to men. Sex differences in mortality and vulnerability to the COVID-19 disease, observed also in Italy (China, 2019; Chen et al., 2020; Wenham et al., 2020), could be associated to different degrees of adherence to quarantine restrictions for gender, with women being more likely vigilant about preventive and avoidant behaviors.

As suggested by findings from previous studies regarding age and gender patterns of risk-taking behaviors (Pawlowski et al., 2008; Cobey et al., 2013), men are more likely to engage in risk-taking behaviors.

The pattern of findings for age is not straightforward. Italian youngest individuals (18–29 years) tended to be least adherent among all the age groups. Subjects aged 30–39 years had significantly lower levels of adherence compared to people aged 50–59 years. People older than 60 years had levels of adherence lower than those of the other age groups, except for the 18- to 29-year age group. Adherence seems to heighten with increasing of age until 59 years, after which the trend surprisingly reversed, with the over-60s reporting to adhere less to the quarantine guidelines, similarly to the 30- to 39-year group. The two extremes of the life span (very young: from 18 to 39 years and seniors: 60+ years) have been found more reluctant to comply with the quarantine guidelines. If this is not a great problem for youngest people, it certainly is for the elderly, who are most particularly at risk of contracting the COVID-19 with fatal consequences. Older age has been reported associated with adverse clinical outcomes, including hospitalization and mortality (Applegate and Ouslander, 2020; Chen et al., 2020). Indeed, in Italy the mean age of the COVID-19 patients who died was 81 years (Remuzzi and Remuzzi, 2020), and case fatality rate was 16% from 60 to 79 years, 19.7% from 80 to 89 years, and 16% for 90 years or older (Livingston and Bucher, 2020). Reasons of this unexpected behavior (Lau et al., 2003; Leung et al., 2004, 2005; Quah and Hin-Peng, 2004; Tang and Wong, 2004) were, in elderly subjects of our sample, the loss of freedom to movement (57.4%) and renouncing to important habits, such as to going to the recreational club (47.4%). It seems that the management of daily time, the loss of real social contacts, and the difficulty accessing to “virtual” interactions via social networks can easily undermine compliance in this age group (Yip et al., 2010; Zhong et al., 2017). In a population where loneliness and isolation have already been described as an epidemic, the impact of even short-term social distancing measures merits careful and urgent study. As regards educational attainment, our findings indicated that increasing education was associated with adherence to quarantine restrictions, with people with high education showing highest scores of adherences among all the groups, in accordance with previous studies (Webster et al., 2020).

As regards marital status, single and widowed people are the least adherent group with quarantine policies in the event of this outbreak. This datum was in line with part of previous literature reporting that ever-married people had more compliance with quarantine policies compared to never-married people (Tang and Wong, 2004; Lau et al., 2007). It is plausible that these people had greater difficulty in relying on or obtaining the assistance of others.

As geographical area of residence, South of Italy showed the highest levels of adherence among all the groups, except for the Islands. Regions of Center of Italy were more likely to adhere with guidelines compared to North-East regions.

This datum is very interesting because Southern and Central regions had recorded minor number of deaths and diagnosed cases (938 and 10,452, 1,720, and 19,059, respectively), compared to North-East and North-West regions (4,582 deaths and 10,452 cases, and 14,652 deaths and 83,971 cases, respectively), which are the most severely affected areas, despite two considerations. First, the Italian Government implemented control measures in the Northern regions, before any other region, and carried out extraordinary efforts to restrict the movement of people at the expense of the Italian economy. Second, a huge number of people – mainly students attending universities in Northern Italy – came back from Po Valley to their families in the South just in the middle of the outbreak, representing potential factor able to accelerate the spreading of the viral infection.

Despite this, the greater spread of contagion was recorded in the North of Italy. Among other factors, the reason may lie also in less adherence of its residents toward the strict self- and forced-quarantine measure, compared to the rest of Italy.

The attitude of greater adherence of the respondents from Southern Italy could challenge cultural stereotypes about the alleged poor civic sense that southerners would have compared to northerners (Viesti, 2013).

As employment status, students showed the significantly lowest mean of adherence among all the other groups, consistent with findings of a previous study (Soud et al., 2009). Consistently with the data collected during this pandemic in China (Zhong et al., 2020), these differences could be ascribed to younger age.

Among occupational groups, healthcare workers showed significantly higher means of adherence compared to students and employed and unemployed people.

The adherence of healthcare workers to all the stringent occupational guidelines in the event of COVID-19 outbreaks is very critical because they are the occupational categories most at risk of contracting the virus during the COVID-19 epidemic (World Health Organization, 2020a). In Italy, because they have not been equipped promptly with self-protective equipment (PPE, such as gown, gloves, N95 masks, goggles, etc.), neither adequate nor sufficient IPC (infection prevention and control) training for respiratory pathogens had worked in life-threatening healthcare settings, with longer duty hours since February 20.

High fatality rates may occur in these health settings if widespread non-adherence to safety measures occurs, also for the risk of contagion and spreading the virus to their families, friends, or colleagues. At time of writing, in Italy around 10% (*n* = 16,050) of health care professionals have become infected, and 121 doctors have died (FNOMCeO, 2020).

Thus, as Remuzzi stated, “Our doctors and nurses are modern heroes in an unexpected war against a difficult enemy” (p.4) (Remuzzi and Remuzzi, 2020). Among healthcare professionals, physicians, nurses, and pharmacists exhibited significantly higher means of adherence to protocols compared with non-frontline health care workers, such as psychologists and rehabilitation therapists. This is also in line with previous experiences from severe acute respiratory syndrome/Middle East respiratory syndrome, showing frontline health professionals constitute a unique risk group compared with professionals working in second-line positions (Gardner and Moallef, 2015; Lee et al., 2018).

A weak but significant positive association was found between the self-reported adherence and the length of quarantine. This datum appears to be in line with the mixed evidences in literature on how the length of prescribed quarantine affects adherence to quarantine protocol (Webster et al., 2020).

People accounted for their compliance with the quarantine order on personal, ethical, social, or legal grounds. The most important reasons for complying were to prevent the spread of COVID-19 (79.8%) and to reduce the risk of transmission to others (75.7%). Also, the trust in government action to control the pandemic (e.g., “I trust the measurements to protect us from contracting COVID-19 are useful,” “the authorities recommend them”) is a crucial factor (40.2%). Importantly, even if the trust in the government is below average, the adherence of our participants to its recommendations is very high.

Differently to previous studies on past epidemics (Webster et al., 2020), ethical reasons were most uncommon with our participants: only the 0.1% say that they complied with quarantine to be “good citizens” or to do their “civic duty.” As well as, only 2.3% of the participants take protective behaviors for social norms toward COVID-19 prevention (e.g., agreement on the statement “other people at home or at work already follow measures to prevent against COVID-19”) or for legal reasons were also cited (“I don’t want problems with the law”) (0.2%).

The reasons for not adhering to quarantine and negative aspects of adhering to quarantine were examined. Obstacles to compliance were in having in biased risk perception (“I don’t think I’m at risk of contagion,” “coronavirus is not so serious”) (0.06% totally), or in attitude to general self-neglect (“I don’t care about my health,” “I never got sick”) (0.06% totally).

Another kind of service needed by those in quarantine is psychosocial support to fight boredom and mute the stigma that could easily undermine compliance (DiGiovanni et al., 2004).

The most common negative experiences associated with the staying home were identified in the boredom deriving from being closed in the house (43.7%); renouncing to important habits (shopping, going to the gym, to the hairdresser/beautician, the recreational clubs, etc.) (74.3%); lacking of being free to movement, such as traveling for leisure or business (71.4%); the lack of a working environment (colleagues, routines, etc.) (29.7%); not being able to do something useful (26.8%); the inability to manage own daily time (25.8%); greater conflict in the family (14.8%); uncertainty about the future consequences (7.5%); forced and prolonged coexistence with unwelcome people (3.2%); and the imposition of such stringent rules (2.5%).

Lengthy confinement of people in their homes could also produce tensions within households that could become dangerous (Bish and Michie, 2010). Microsocial effects, such as ostracism within the family, questioning of the professional activity, and conflicts following selective sharing of information with relatives about risk exposure, can have an impact on intrafamilial relationships, when power relationships or preexisting conflicts put some members at a disadvantage (DiGiovanni et al., 2004; Johal, 2009).

The strength of this study lies in its large sample recruited during a critical period, the early stage of the COVID-19 outbreak in Italy. Nevertheless, the predominance of women and undergraduate students (who are generally young and perhaps have fewer responsibilities than adults who are employed full-time) and the small number of healthcare workers limit our ability to generalize these findings to a wider population. In addition, the sample recruitment approach through social media channels, due to the exceptional pandemic conditions, can have permitted the participation of only those who had a computer and computer literacy (probably excluding middle–old/old–old participants). However, features of our sample are similar to the others reported in already available studies carried out in the Italian context during the COVID-19 emergency (Barari et al., 2020; Mazza et al., 2020; Moccia et al., 2020). Another limitation of our study is related to the adoption of self-reported and not already validated questionnaires. Moreover, this survey has been translated into different languages with the aim of evaluating the impact of pandemic in other countries. It should also be noted that, given the public salience of these measures, it is likely that social desirability biased the self-reporting behaviors. Future studies should validate the compliance rates shown here for real-world observational data. For example, the registration in the questionnaire of possible arrests or sanctions in case of skipping the quarantine could provide objective measures of the adherence, as well as an implicit degree of responsibility of the population. Similarly, future research to understand how social (Saggino et al., 2017), religious (Carlucci et al., 2015), and economic factors, as well as personality factors (Innamorati et al., 2014), affect compliance with quarantine will be helpful in planning for future public health emergencies. Because there are likely to be cultural and societal differences in responses to a pandemic, some broad conclusions can be drawn from the evidence identified, but their applicability is likely to vary across country. Thus, caution should be given when generalizing the results of this study to other countries. International comparisons are therefore also greatly warranted.

## Conclusion

Findings from previous researches suggested that some countries with quarantine still had problems with compliance, as evidenced by increasing fines and arrest penalties (Blendon et al., 2006). To increase compliance, public health authorities need to plan in advance. They should prepare trusted spokespeople to explain to the public the steps that need to be taken to halt the spread of the disease and stress the need for compliance and take the Italian model as example. With full population compliance with quarantine policies, the critical battlefront of the COVID-19 epidemic would shift to effective hospital infection control. As Saglietto stated, “We urge all countries to acknowledge the Italian lesson and to immediately adopt very restrictive measures to limit viral diffusion, ensure appropriate health system response, and reduce mortality, which appears to be higher than previously estimated, with a crude case-fatality rate of almost 4%” (p.1110) (Saglietto et al., 2020).

In this study, the perception of risk about contracting or spreading disease, associated with adherence to quarantine guidelines in Italian community, was also analyzed. To date, there are no studies on risk perception of infectious diseases COVID-19 in Europe. Therefore, this study makes an important contribution to the field and could be helpful for these countries that are hesitant to apply the quarantine protocols or which nowadays are evaluating its effects, due to a later spread of the contagion.

## Data Availability Statement

The raw data supporting the conclusions of this article will be made available by the authors, without undue reservation.

## Ethics Statement

The studies involving human participants were reviewed and approved by the Department of Psychological Sciences, Health and Territory, University of Studies “G. d’Annunzio” Chieti–Pescara, Italy, Review Board. The patients/participants provided their written informed consent to participate in this study.

## Author Contributions

MB and LC designed the study and drafted the manuscript. LC conducted the statistical analyses. ID’A collected the data. MB, ID’A, and LC interpreted the data. All authors contributed toward revising the manuscript and agreed to be accountable for all aspects of the work.

## Conflict of Interest

The authors declare that the research was conducted in the absence of any commercial or financial relationships that could be construed as a potential conflict of interest.

## Percezione Del Rischio *Risk Perception*

(A) Percezione del Rischio/Risk Perception (8-item);
  • Penso che contrarrò il COVID-19 se entro in contatto con un paziente affetto da COVID-19.
    I think that I will contract COVID-19 if I come into contact with a COVID-19 patient.
  • Penso che potrei contrarre il COVID-19 anche se non entro in contatto con un paziente affetto da COVID-19.
    I think that I might contract COVID-19 even if I do not come into contact with a COVID-19 patient.
  • La mia salute sarà gravemente compromessa se contraggo il COVID-19.
    My health will be severely damaged if I contract COVID-19.
  • Penso che il COVID-19 è molto più grave rispetto alle altre malattie respiratorie.
    I think that COVID-19 in more severe than any other respiratory diseases.
  • Anche se mi ammalassi di un’altra malattia, non mi recherei in ospedale a causa del COVID-19.
    Even if I fall ill with another disease, I will not go to hospital because of COVID-19.
  • Il COVID-19 causerà seri danni alla mia comunità.
    COVID-19 will inflict serious damage to my community.
  • Il COVID-19 si diffonderà nuovamente in Italia un giorno o l’altro.
    COVID-19 may spread in Italy again someday.
  • Penso che potrei contrarre il COVID-19 anche se non entro in contatto con una persona affetta da COVID-19 perché potrebbe essere asintomatica e ignara.
    I think I will contact COVID-19 even if I do not come into contact with a COVID-19 patient because he/she might be lack of symptoms or suspect nothing of them.
(B) Ansia e Suscettibilità/Anxiety and Susceptibility (3 item);
  • Quanto sei preoccupato di contrarre il COVID-19?
    How worried are you about getting COVID-19?
  • Che probabilità hai di contrarre il COVID-19 se non segui le misure preventive?
    What are your chances of getting COVID-19 if you do not follow preventive measures adopted?
  • Se non dovessi essere vaccinato che probabilità avrai nel contrarre il COVID-19 entro questo anno?
    If you don’t get vaccinated, what are your chances of getting COVID-19 within this year?
(C) Intenzione nel seguire le misure preventive/Intention to carry out the quarantine guidelines (1 item);
  • Hai intenzione di seguire le misure preventive?
    Do you intend to follow the preventive measures?
(D) Perception of seriousness (1 item);
  • Quanto pensi che sia grave l’emergenza da coronavirus sulla salute mondiale?
    How severe do you think the coronavirus emergency on global health is?
(E) Fattori motivanti / ostacolanti che determinano la volontà di attuare le misure preventive
  *Motivating/hindering factors that determine the willingness to carry out preventive measures* (1 item).
  • Perchè esegui le misure preventive?
    Why are you taking preventive measures?
    ○ Sono spesso malato.
      I’m often sick.
    ○ Il COVID-19 può essere pericoloso.
      COVID-19 can be dangerous.
    ○ Mi sento responsabile della mia salute.
      I feel responsible for my health.
    ○ Penso di essere a rischio COVID-19.
      I think I’m at risk COVID-19.
    ○ Voglio prevenire il diffondersi del COVID-19.
      *I want to prevent the spread of COVID-19*.
    ○ Non voglio trasmettere il COVID-19 alle persone a me vicine.
      I don’t want to transmit COVID-19 to people close to me.
    ○ Confido che le misure siano utili.
      I trust the preventive measures are helpful.
    ○ Le autorità le raccomandano.
      authorities recommend them.
    ○ Potrei pentirmene dopo, se non le eseguo.
      I might regret it later if I don’t take them.
    ○ Altre persone in casa o al lavoro, le seguono già.
      Other people at home or at work are following them already.
    ○ Altro…
      Other…

## Aderenza *Adherence*

• Lavi le mani con acqua e sapone o con gel a base alcolica?
  Did you wash your hands using soapy water or an alcohol-based solution?
• Nei contatti sociali, mantieni una distanza di almeno un metro?
  When you had a social interaction, did you keep a distance no closer than six feet from the others?
• Starnutisci e/o tossisci in un fazzoletto evitando il contatto delle mani con le secrezioni respiratorie?
  Did you sneeze and/or cough in a tissue or elbow, preventing the hands from coming into contact with respiratory secretion?
• Eviti l’uso promiscuo di bottiglie e bicchieri?
  Did you avoid sharing bottles and cups for drinking?
• Eviti di toccare occhi, naso e bocca con le mani?
  Did you avoid touching your face, nose and mouth with your hands?
• Copri la bocca e il naso, se starnutisci e/o tossisci?
  Did you cover your mouth or nose when you sneeze and/or cough?
• Eviti strette di mano e abbracci?
  Did you avoid handshaking and hugging?
• Pulisci le superfici di casa o ufficio con disinfettante a base di cloro o alcol?
  Did you clean your home or office surfaces with disinfectant wipes or spray?
• Eviti assembramenti in luoghi pubblici o aperti al pubblico?
  Did you avoid gatherings in public places and avoid hosting gatherings in your home?
• Svolgi sport e/o attività motorie all’aperto da solo?
  Did you participate in outdoor sport and/or physical activities alone?
• Usi la mascherina?
  Did you wear a protective mask?
• Prendi farmaci antivirali e antibiotici?
  Did you take antiviral drugs or antibiotics?

## References

[B1] Adams J. (1995). *Risk.* London, UK: UCL Press.

[B2] Ajilore K. Atakiti I. Onyenankeya K. (2017). College students’ knowledge, attitudes and adherence to public service announcements on Ebola in Nigeria: Suggestions for improving future Ebola prevention education programmes. *Health Educat. J.* 76 648–660. 10.1177/0017896917710969

[B3] Ajzen I. (1991). The theory of planned behavior. *Organizat. Behav. Hum. Dec. Proc.* 50 179–211.

[B4] Applegate W. B. Ouslander J. G. (2020). COVID-19 Presents High Risk to Older Persons. *J. Am. Geriatr. Soc.* 68:681. 10.1111/jgs.16426 32154911 PMC7166412

[B5] Barari S. Caria S. Davola A. Falco P. Fetzer T. Fiorin S. (2020). Evaluating COVID-19 Public Health Messaging in Italy: Self-Reported Compliance and Growing Mental Health Concerns. *medRxiv* 2020:20042820 10.1101/2020.03.27.20042820

[B6] Bensimon C. M. Upshur R. E. (2007). Evidence and effectiveness in decisionmaking for quarantine. *Am. J. Pub. Health* 97 (Suppl. 1), S44–S48. 17413076 10.2105/AJPH.2005.077305 PMC1854977

[B7] Bish A. Michie S. (2010). Demographic and attitudinal determinants of protective behaviours during a pandemic: a review. *Br. J. Health Psychol.* 15 797–824. 10.1348/135910710x485826 20109274 PMC7185452

[B8] Blendon R. J. DesRoches C. M. Cetron M. S. Benson J. M. Meinhardt T. Pollard W. (2006). Attitudes Toward The Use Of Quarantine In A Public Health Emergency In Four Countries: The experiences of Hong Kong, Singapore, Taiwan, and the United States are instructive in assessing national responses to disease threats. *Health Affair.* 25(Suppl.1), W15–W25. 10.1377/hlthaff.25.w15 16434437

[B9] Brewer N. T. Chapman G. B. Gibbons F. X. Gerrard M. McCaul K. D. Weinstein N. D. (2007). Meta-analysis of the relationship between risk perception and health behavior: the example of vaccination. *Health Psychol.* 26 136–145. 10.1037/0278-6133.26.2.136 17385964

[B10] Carlucci L. Tommasi M. Balsamo M. Furnham A. Saggino A. (2015). Religious Fundamentalism and Psychological Well-Being: An Italian Study. *J. Psychol. Theol.* 43 23–33. 10.1177/009164711504300103

[B11] Chen N. Zhou M. Dong X. Qu J. Gong F. Han Y. (2020). Epidemiological and clinical characteristics of 99 cases of 2019 novel coronavirus pneumonia in Wuhan. *Chin. Descript. Stud. Lancet* 395 507–513. 10.1016/s0140-6736(20)30211-7 PMC7135076 32007143

[B12] China C. (2019). Novel Coronavirus Pneumonia Emergency Response Epidemiology Team. *Vital Surve. Epidemiol. Character. Outbreak.* 2020 145–151.

[B13] CobeyK. D.LaanF.StulpG.BuunkA. P.PolletT. V. J. E. P. (2013). Sex differences in risk taking behavior among Dutch cyclists. *Evol. Psychol.* 11 350–64.23674522

[B14] Cohen J. (1988). *Statistical power analysis for behavioral sciences*, 2nd Edn Hillsdale, NJ: Erlbaum.

[B15] DiGiovanni C. Conley J. Chiu D. Zaborski J. (2004). Factors influencing compliance with quarantine in Toronto during the 2003 SARS outbreak. *Biosec. Bioterr. Biodef. Strat. Pract. Sci.* 2 265–272. 10.1089/bsp.2004.2.265 15650436

[B16] Ferguson N. Laydon D. Nedjati-Gilani G. Imai N. Ainslie K. Baguelin M. (2020). *Imperial College COVID-19 Response Team.* United Kingdom: Imperial College London.

[B17] FNOMCeO (2020). *Elenco dei Medici caduti nel corso dell’epidemia di Covid-19.* Available online at: https://portale.fnomceo.it/elenco-dei-medici-caduti-nel-corso-dellepidemia-di-covid-19/ (accessed May 1, 2020).

[B18] Gardner P. J. Moallef P. (2015). Psychological impact on SARS survivors: Critical review of the English language literature. *Canad. Psychol. Psychol. Canad.* 56:123 10.1037/a0037973

[B19] Gernhart G. (1999). A forgotten enemy: PHS’s fight against the 1918 influenza pandemic. *Publ. Health Rep.* 114:559. 10.1093/phr/114.6.559 10670624 PMC1308540

[B20] Government of Italy (2020a). *Decree of the president of the Council of Ministers.* Available online at: https://www.gazzettaufficiale.it/eli/id/2020/03/09/20A01558/sg (accessed May 1, 2020).

[B21] Government of Italy (2020b). *Decree of the president of the Council of Ministers^∗^.* Available online at: https://www.gazzettaufficiale.it/eli/id/2020/03/11/20A01605/sg (accessed May 1, 2020).

[B22] Graves K. D. Huerta E. Cullen J. Kaufman E. Sheppard V. Luta G. (2008). Perceived risk of breast cancer among Latinas attending community clinics: risk comprehension and relationship with mammography adherence. *Cancer Causes* 19 1373–1382. 10.1007/s10552-008-9209-7 18704716 PMC4309545

[B23] Greenberger M. (2018). Better Prepare Than React: Reordering Public Health Priorities 100 Years After the Spanish Flu Epidemic. *Am. J. Publ. Health* 108 1465–1468. 10.2105/ajph.2018.304682 30252520 PMC6187800

[B24] Innamorati M. Lester D. Balsamo M. Erbuto D. Ricci F. Amore M. (2014). Factor validity of the beck hopelessness scale in Italian medical patients. *J. Psychopathol. Behav. Assess.* 36, 300–307. 10.1007/s10862-013-9380-3

[B25] Johal S. S. (2009). Psychosocial impacts of quarantine during disease outbreaks and interventions that may help to relieve strain. *N Z Med. J.* 122(1296), 47–52. 19652680

[B26] Lau J. Kim J. H. Tsui H. Y. Griffiths S. (2007). Anticipated and current preventive behaviors in response to an anticipated human-to-human H5N1 epidemic in the Hong Kong Chinese general population. *BMC Infect. Dis.* 7:18. 10.1186/1471-2334-7-18 17359545 PMC1845150

[B27] Lau J. Yang X. Tsui H. Kim J. (2003). Monitoring community responses to the SARS epidemic in Hong Kong: from day 10 to day 62. *J. Epidemiol. Commun. Health* 57 864–870. 10.1136/jech.57.11.864 14600111 PMC1732318

[B28] Lee S. M. Kang W. S. Cho A.-R. Kim T. Park J. K. (2018). Psychological impact of the 2015 MERS outbreak on hospital workers and quarantined hemodialysis patients. *Comprehens. Psych.* 87 123–127. 10.1016/j.comppsych.2018.10.003 30343247 PMC7094631

[B29] Leung G. Ho L.-M. Chan S. K. Ho S.-Y. Bacon-Shone J. Choy R. Y. (2005). Longitudinal assessment of community psychobehavioral responses during and after the 2003 outbreak of severe acute respiratory syndrome in Hong Kong. *Clin. Infect. Dis.* 40 1713–1720. 10.1086/429923 15909256

[B30] Leung G. Quah S. Ho L. Ho S. Hedley A. Lee H. (2004). A tale of two cities: community psychobehavioral surveillance in Hong Kong and Singapore during the severe acute respiratory syndrome epidemic. *Infect Contr. Hosp. Epidemiol.* 25 1033–1041. 10.1086/502340 15636289

[B31] Livingston E. Bucher K. (2020). Coronavirus disease 2019 (COVID-19) in Italy. *JAMA* 32 1335–1133. 10.1001/jama.2020.4344 32181795

[B32] Maroldi M. A. C. da Silva, Felix A. M. Dias A. A. L. Kawagoe J. Y. Padoveze M. C. (2017). Adherence to precautions for preventing the transmission of microorganisms in primary health care: a qualitative study. *BMC Nurs.* 16:49. 10.1186/s12912-017-0245-z 28919838 PMC5594588

[B33] Mazza C. Ricci E. Biondi S. Colasanti M. Ferracuti S. Napoli C. (2020). A nationwide survey of psychological distress among italian people during the COVID-19 pandemic: Immediate psychological responses and associated factors. *Int. J. Environ. Res. Publ. Health* 17:3165. 10.3390/ijerph17093165 32370116 PMC7246819

[B34] Moccia L. Janiri D. Pepe M. Dattoli L. Molinaro M. De Martin V. (2020). Affective temperament, attachment style, and the psychological impact of the COVID-19 outbreak: an early report on the Italian general population. *Brain Behav. Immun.* 87 75–79. 10.1016/j.bbi.2020.04.048 32325098 PMC7169930

[B35] Murphy K. R. Myors B. Wolach A. (2014). *Statistical power analysis: A simple and general model for traditional and modern hypothesis tests, 4th ed.* New York, NY: Routledge/Taylor & Francis Group.

[B36] Olejnik S. Algina J. (2003). Generalized eta and omega squared statistics: measures of effect size for some common research designs. *Psychol. Methods* 8 434–447. 10.1037/1082-989x.8.4.434 14664681

[B37] Pawlowski B. Atwal R. Dunbar R. I. M. (2008). Sex Differences in Everyday Risk-Taking Behavior in Humans. *Evolut. Psychol.* 6 29–42. 10.1177/147470490800600104

[B38] Quah S. R. Hin-Peng L. (2004). Crisis prevention and management during SARS outbreak, Singapore. *Emerg. Infect. Dis.* 10 364–368. 10.3201/eid1002.030418 15030714 PMC3322925

[B39] Remuzzi A. Remuzzi G. (2020). COVID-19 and Italy: what next? *Lancet* 395 1225–1228. 10.1016/s0140-6736(20)30627-9 32178769 PMC7102589

[B40] Richardus J. Korfage I. French J. van Steenbergen S. Das H. Voeten H. (2015). “Effective communication in outbreak management (ECOM),”in *Preconference organized at European Scientific Conference on Applied Infectious Disease Epidemiology (ESCAIDE)*, (Europe: ESCAIDE).

[B41] Rogers R. W. (1975). A protection motivation theory of fear appeals and attitude change1. *J. Psychol.* 91 93–114. 10.1080/00223980.1975.9915803 28136248

[B42] Rosenstock I. M. (1974). The health belief model and preventive health behavior. *Health Educat. Monogr.* 2 354–386. 10.1177/109019817400200405

[B43] Saggino A. Carlucci L. Sergi M. R. D’Ambrosio I. Fairfield B. Cera N. (2017). A validation study of the psychometric properties of the Other as Shamer Scale–2. *SAGE Open* 7:2158244017704241 10.1177/2158244017704241

[B44] Saglietto A. D’Ascenzo F. Zoccai G. B. De Ferrari G. M. (2020). COVID-19 in Europe: the Italian lesson. *Lancet* 395 1110–1111. 10.1016/s0140-6736(20)30690-5 32220279 PMC7118630

[B45] Schabas R. (2004). Severe acute respiratory syndrome: Did quarantine help? *Canad. J. Infect. Dis. Med. Microbiol.* 15 204–204. 10.1155/2004/521892 18159492 PMC2094974

[B46] Soud F. Cortese M. Curns A. Edelson P. Bitsko R. Jordan H. (2009). Isolation compliance among university students during a mumps outbreak, Kansas 2006. *Epidemiol. Infect.* 137 30–37. 10.1017/s0950268808000629 18387216

[B47] Spina S. Marrazzo F. Migliari M. Stucchi R. Sforza A. Fumagalli R. (2020). The response of Milan’s Emergency Medical System to the COVID-19 outbreak in Italy. *Lancet* 395 e49–e50. 32119824 10.1016/S0140-6736(20)30493-1 PMC7124430

[B48] Storholm E. D. Volk J. E. Marcus J. L. Silverberg M. J. Satre D. D. (2017). Risk perception, sexual behaviors, and PrEP adherence among substance-using men who have sex with men: a qualitative study. *Prevent. Sci.* 18 737–747. 10.1007/s11121-017-0799-8 28578516 PMC5558785

[B49] Sutton S. (1987). Social-psychological Approaches to Understanding Addictive Behaviours: attitude-behaviour and decision-making models. *Br. J. Addict.* 82 355–370. 10.1111/j.1360-0443.1987.tb01492.x 3472583

[B50] Tang C. S.-K. Wong C.-Y. (2004). Factors influencing the wearing of facemasks to prevent the severe acute respiratory syndrome among adult Chinese in Hong Kong. *Prevent. Med.* 39 1187–1193. 10.1016/j.ypmed.2004.04.032 15539054 PMC7133369

[B51] Viesti G. (2013). *Il Sud vive sulle spalle dell’Italia che produce.* Bari: Laterza.

[B52] Webster R. K. Brooks S. K. Smith L. E. Woodland L. Wessely S. Rubin G. J. (2020). How to improve adherence with quarantine: Rapid review of the evidence. *Publ. Health* 182 163–169. 10.1016/j.puhe.2020.03.007 32334182 PMC7194967

[B53] Weinstein N. D. (1993). Testing four competing theories of health-protective behavior. *Health Psychol.* 12 324–333. 10.1037/0278-6133.12.4.324 8404807

[B54] Weinstein N. D. (2000). Perceived probability, perceived severity, and health-protective behavior. *Health Psychol.* 19 65–74. 10.1037/0278-6133.19.1.65 10711589

[B55] Wenham C. Smith J. Morgan R. (2020). COVID-19: the gendered impacts of the outbreak. *Lancet* 395 846–848. 10.1016/s0140-6736(20)30526-2 32151325 PMC7124625

[B56] Witte K. (1992). Putting the fear back into fear appeals: The extended parallel process model. *Commun. Monogr.* 59 329–349. 10.1080/03637759209376276

[B57] World Health Organization (2020a). *Coronavirus disease 2019 (COVID-19): situation report.* Switzerland: World Health Organization, 82.

[B58] World Health Organization. (2020b). *Virtual press conference on COVID-19.* Switzerland: World Health Organization.

[B59] Yip P. S. Cheung Y. Chau P. H. Law Y. (2010). The impact of epidemic outbreak: the case of severe acute respiratory syndrome (SARS) and suicide among older adults in Hong Kong. *Cris. J. Cris. Intervent. Suic. Prevent.* 31 86–92. 10.1027/0227-5910/a000015 20418214

[B60] Zhong B.-L. Chen S.-L. Tu X. Conwell Y. (2017). Loneliness and cognitive function in older adults: Findings from the Chinese longitudinal healthy longevity survey. *J. Gerontol. Ser. B* 72 120–128. 10.1093/geronb/gbw037 27013536 PMC5156491

[B61] Zhong B.-L. Luo W. Li H.-M. Zhang Q.-Q. Liu X.-G. Li W.-T. (2020). Knowledge, attitudes, and practices towards COVID-19 among Chinese residents during the rapid rise period of the COVID-19 outbreak: a quick online cross-sectional survey. *Int. J. Biol. Sci.* 16 1745–1752. 10.7150/ijbs.45221 32226294 PMC7098034

